# Estimating Effects of Sea Level Rise on Benthic Biodiversity and Ecosystem Functioning in a Large Meso-Tidal Coastal Lagoon

**DOI:** 10.3390/biology12010105

**Published:** 2023-01-10

**Authors:** Olivia Dixon, Johanna Gammal, Dana Clark, Joanne I. Ellis, Conrad A. Pilditch

**Affiliations:** 1 School of Science, The University of Waikato, Hamilton 3240, New Zealand; 2 Cawthron Institute, Nelson 7010, New Zealand; 3 School of Science, The University of Waikato, Tauranga 3110, New Zealand

**Keywords:** sea-level rise, estuaries, intertidal area loss, benthic macrofauna, functional groups, Aotearoa New Zealand

## Abstract

**Simple Summary:**

Estuaries are among the world’s most productive ecosystems, but due to their location between land and open sea, they are affected by many anthropogenic pressures, including the consequences of climate change. A rising sea level is one major consequence, which will affect both humans and ecosystems, especially in estuaries with extensive intertidal habitats. There is, however, a lack of knowledge regarding the ecological implications of losing intertidal habitats. Therefore, we investigated how seafloor macrofauna communities and their contribution to ecosystem functioning may change due to rising sea levels. Based on a spatially extensive dataset on macrofauna and environmental variables, we identified three main community groups representing intertidal, shallow subtidal, and deep subtidal habitats. Functional trait analysis indicated low functional redundancy for a key intertidal suspension-feeding bivalve (*Austrovenus stutchburyi*) and the lack of a shallow subtidal functional replacement should intertidal habitats become inundated (i.e., become shallow subtidal habitats). These findings thus strongly suggest that sea level rise and the associated environmental changes will modify the seafloor macrofauna communities in estuaries, and subsequently, the ecosystem functions that they influence will be altered.

**Abstract:**

Estuaries are among the world’s most productive ecosystems, but due to their geographic location, they are at the forefront of anthropogenic pressures. Sea level rise (SLR) is one major consequence of climate change that poses a threat to estuaries with extensive intertidal habitats. The ecological implications of intertidal habitat loss have been largely overlooked despite their likely significance. We aimed to address this knowledge gap by investigating how benthic macroinvertebrate communities and their contributions to ecosystem function are likely to respond to SLR. Based on a spatially extensive dataset (119 sites) from a large coastal lagoon, depth, sediment chlorophyll concentrations, mud content, and average current speed were identified as the main drivers of community compositional turnover. Shifts in benthic community structure and associated functional implications were then evaluated using depth as a proxy for SLR. Three main macrofaunal groups representing intertidal, shallow subtidal, and deep subtidal habitats were identified. Functional trait analysis indicated low functional redundancy for a key intertidal suspension-feeding bivalve (*Austrovenus stutchburyi*) and the lack of a shallow subtidal functional replacement should intertidal habitats become inundated. These findings strongly suggest SLR and the associated environmental changes will alter estuarine macroinvertebrate communities, with implications for future ecosystem function and resilience.

## 1. Introduction

Estuaries comprise some of the world’s most productive and widespread ecosystems and deliver vital ecosystem services used by humans around the globe [1,2]. The ecosystem services provided by these dynamic and complex environments include food provision, nutrient and carbon processing, coastal protection, and recreational activities [3,4,5]. Furthermore, the production from estuaries is fueling the wider coastal food webs [6], contributing to valuable nursery grounds for fish [7], and important foraging habitats for sea birds [8]. This high level of functionality is underpinned by soft-sediment benthic communities and the processes they regulate, e.g., [9,10]. Thus, understanding how these ecosystems respond to changing environmental conditions is critical for understanding broader-scale changes within coastal ecosystems.

Estuarine habitats, situated at the interface between the land and the sea, are at the forefront of localized (within catchment) anthropogenic stressors such as sedimentation, eutrophication, and pollution, often resulting from excess inputs of terrestrial sediment, nutrients, and contaminants [11,12]. There is a rich body of literature describing the impacts of these stressors on benthic communities and associated ecosystem functioning [13,14,15,16,17]. However, these ecosystems are also vulnerable to global scale climate change, in particular sea level rise (SLR, amongst others), of which the ecological impacts have received considerably less attention (but see, e.g., [18,19,20,21,22]).

Coastal barrier lagoons are a common type of estuary globally, and they are characterized by deeper, permanently submerged channels and extensive intertidal flats [23]. Such systems are particularly vulnerable to SLR because even a small increase in water depth can result in large reductions in intertidal areas. As an example, using a one-meter SLR scenario, Mangan et al. [18] estimated up to an 80% loss of intertidal area within 12 Aotearoa New Zealand estuaries. Under current predictions, the global mean sea level is estimated to rise between 0.3 m and 2 m by 2100 (compared to 2000 levels), following lowest and highest global greenhouse gas emission pathways, respectively [24,25]. While extensive research has focused on physical impacts such as changes to coastal geomorphology [26,27,28], including quantifying the loss of intertidal areas [8,19,29], the ecological impacts on soft sediment ecosystems have received much less attention.

The ecological changes (i.e., shifts in biodiversity and ecosystem function) that are likely to arise in response to an altered coastal environment (i.e., deeper water column, steeper slopes, and changed sedimentary environment) are not well documented but see e.g., [18,19,20,21,22]. Given the important role macro-benthic communities play in regulating ecosystem functions, understanding how communities might change with SLR will provide some insight into how ecosystem functions and services might be impacted. Here we make use of an extensive data set from a large shallow coastal lagoon to explore how macro-benthic community composition may be altered with SLR. By considering the functional traits of the macrofaunal species, we also explore whether shifts in community composition translate to potential shifts in function.

We focus on macrofaunal communities because their significance in sustaining valued ecosystem services is well recognized. Through bioturbation and feeding activities, these organisms enhance ecosystem functionality contributing to primary and secondary production, nutrient cycling/processing, sediment stabilization, habitat formation, and carbon sequestration, e.g., [30,31,32,33,34,35]. However, not all species contribute equally, with some making a disproportionate contribution. For example, evidence indicates that larger individuals play a greater role in facilitating solute fluxes (e.g., nitrogen and oxygen) and maintaining community structure than smaller ones, directly due to their body mass and indirectly through the larger impact of their bioturbation or generation of habitat [36]. Taxonomic groups distinguished by certain functional traits (e.g., bioturbating bivalves), therefore, often hold unique roles in ecosystem functionality. This suggests that the functional group diversity response to altered environmental conditions should be considered, in addition to changes in species diversity.

Grouping species based on their functional traits versus focusing on the roles held by individual species is increasingly widespread in community ecology [37]. In marine sediments, loss of functional diversity can impact important biogeochemical processes, including oxygen and nutrient fluxes [38], thus having flow-on effects on overall ecosystem function [39]. Functional redundancy is usually determined by the number and abundance of species sharing similar traits and, therefore, carrying out similar functions [40]. Exploring functional trait diversity allows us to gauge the resilience (determined by the degree of functional redundancy) associated with functional groups and, therefore, the functions regulating important processes such as primary production and nutrient cycling [41]. For example, functional groups that possess a high level of resilience include those with a greater number of species that can persist under varying stressors, such that a loss of an individual species will not necessarily mean loss of the key functions and the respective ecosystem services they contribute to [40,42,43]. Thus, reinforcing the importance of exploring how both species and functional group diversity may shift with rising anthropogenic stressors.

The central objective of this study was to explore the potential implications of SLR on macroinvertebrate community structure and ecosystem functioning within an estuarine setting. Due to the long-time scales associated with climate change, we used space as a proxy for time [44]. We aimed to reveal the response of community structure to shifts in water column depth and the influence of additional environmental factors that are also expected to shift with SLR. Additionally, using Gradient Forest analysis, we investigated if there was evidence of thresholds along environmental gradients where disproportionately greater shifts in community structure (represented by community turnover) occurred. To further understand the ecosystem-level consequences of changes in community structure, we also assessed the response of functional group community structure to shifts in water column depth (and the respective environmental characteristics). Analysis was based on a comprehensive dataset entailing both biological and environmental data (including water column depth) collected in a large barrier-enclosed coastal lagoon.

## 2. Materials and Methods

### 2.1. Study Area

**Figure 1:**
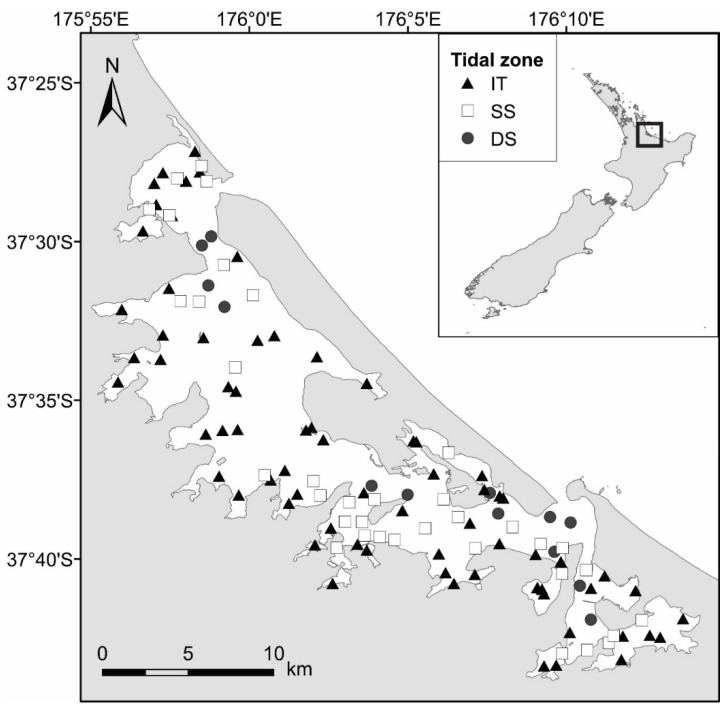
Location of Tauranga Harbor on the northeast coast of New Zealand (insert) and sample site locations within the harbor. The symbols indicate each site’s tidal zone assignation; intertidal (IT), shallow subtidal (SS), and deep subtidal (DS).

Data used for this research were collected within Tauranga Harbor (37°40′ S, 176°10′ E; Figure 1) on the north-eastern coast of Aotearoa, New Zealand’s North Island. Tauranga Harbor is characterized as a large (~200 km^2^), shallow (<10 m depth, mean depth ~3 m), barrier-enclosed estuarine lagoon [45,46]. The harbor has an extensive intertidal area constituting approximately 66% of the estuary [45] and experiences a semi-diurnal tidal cycle with a tidal range of up to 2 m [47]. The harbor catchment is extensive (~1300 km^2^) and includes horticultural, agricultural, and urban land, where water runs from these landscapes into the large estuary. The estuary is well-flushed and vertically well-mixed (tidal and wind mixing), but there is some spatial variation in salinity within the estuary ranging from 28 to 34 [48]. The relative coastal sea level is estimated to have risen at a rate of 2.3 ± 0.26 mm yr^−1^ measured outside Tauranga Harbor over the period of 1974-2020 [49]. The current sea level rise median projections for 2100 according to IPCC SSP1-2.6 and SSP5-8.5 scenarios within the Tauranga Harbor areas vary between 0.3 and 1 m when also taking into account the vertical land movement [50].

### 2.2. Data Acquisition

Data were acquired and combined from two ecological surveys. The first focused primarily on intertidal habitats and was conducted between December 2011 and February 2012 (austral summer) and spanned 75 sites throughout the harbor [16,51]. The second was a subtidal survey carried out between March and May 2016 (late austral summer/autumn) and included 44 sites [52]. Sampling locations were selected to ensure a broad range of environmental gradients were represented, aiming to cover the full spatial extent and depth range (up to 9 m corrected to chart datum) of the harbor. Although these data sets were collected in different years, we believe they can be combined because the large number of sites included in the analysis means that the spatial variation in community structure (i.e., entire harbor) is likely to be greater than any between-year variation.

### 2.3. Environmental Variables

An array of the water column and sedimentary variables were obtained at each site, but only variables measured in both surveys were included in this analysis (Supplementary Table S1). The sampling design and methods were consistent with Aotearoa, New Zealand’s standardized Estuary Monitoring Protocol [53]. At each site, sediment samples were collected with cores 20 mm dia. and 20 mm deep (n = 10 and 6 cores per site within the intertidal survey and the subtidal survey, respectively). For both surveys, the replicates were composited into a single sample, and the sediment was analyzed for grain size, chlorophyll *a* (Chl *a*), nutrient content (total phosphorus TP; total nitrogen TN), organic content (OM, measured by loss on ignition), and heavy metals (lead Pb; zinc Zn; copper Cu) (see Supplementary Table S2 for extraction methods and further details in Ellis et al. [54]). Current speeds were estimated for the coordinates of each site from the Estuary Transport Module [55], where average and maximum values were obtained for this study. Chart datum (CD) depths for each site were determined by subtracting 1.08 m from mean sea level (MSL) values (MSL to CD conversion published by LINZ [Land Information New Zealand]) obtained from a hydrodynamic model grid developed by de Ruiter et al. [56] that incorporates LiDAR data, multibeam survey measurements, and LINZ bathymetric data.

### 2.4. Macrofauna Data

Three replicate core (13 cm diameter, 15 cm deep) samples were taken at each site and sieved on a 0.5 mm mesh to obtain the macrofauna. The macrofauna was preserved in ethanol (70%), counted, and identified to the lowest attainable taxonomic resolution (usually species). Taxa identifications were performed by experts and based on relevant guides (e.g., New Zealand Coastal Marine Invertebrates [57], National Institute of Water and Atmospheric Research (NIWA) Invertebrate Collection [58]) and consultation with taxonomic experts at NIWA. The intertidal and subtidal benthic macrofauna survey datasets were combined, and the taxonomic resolution was standardized to be consistent across surveys. Where individual taxa counts were low (<10 individuals across the combined data set) and the taxonomic resolution was poor (e.g., higher than class), the taxa groups were removed from further analysis (in total, <10 individuals were removed). Counts of larvae and juveniles were also removed from the dataset to reduce any influence of recruitment events on the statistical models. Site averages for macrofauna abundance data (i.e., average abundance per core) were calculated and used for all analyses.

### 2.5. Functional Group Assignment

In order to assess the prospective implications of environmental change on ecosystem function, each taxon was assigned to one of the 26 functional groups developed by Greenfield et al. [59]. The functional groups considered a range of functional traits representing life history, physical morphology, and behavioral characteristics that influence ecosystem functioning and stability in estuarine ecosystems (Table 1). In this study, as the taxonomic resolution was not always to species level, in the case where taxa can exhibit many attributes of a trait (e.g., different species from the family Spionidae qualified for different functional groups), the most dominant attribute of a trait was assigned as the functional group.

### 2.6. Statistical Analyses

#### 2.6.1. Determining Critical Points of Compositional Turnover along Key Environmental Gradients (Gradient Forest Modelling)

To reveal how macroinvertebrate community structure changed along environmental gradients, Gradient Forest (GF) modeling was employed [60,61]. GF identifies critical points along environmental gradients where large shifts in rates of benthic macroinvertebrate compositional turnover occur [60]. GF models allow for the identification of compositional turnover thresholds by aggregating regression-tree-based Random Forest (RF) models. Species considered rare (≤3 occurrences across all 119 sites) were additionally excluded from GF analysis as models are constrained by limited data. The three sites where depth exceeded 6 m were also removed from GF models as there were not enough data to adequately model species turnover beyond this depth. Two key processes are undertaken for GF modeling. The first process uses an extension R package, “extendedForest” [62], which calls on the R package “randomForest” to fit an ensemble of RF models for the input species. These RF models describe the relationship between the species distribution and a set of environmental variables. The second process uses the R package “gradientForest” to aggregate all of the individual split points determined from these models, estimating the most important points of species turnover along each environmental gradient to provide a measure of compositional turnover that represents the entire community.

RF models [61] are a flexible and robust way of modeling non-linear predictor-response relationships. The RF models for individual species are built based on an ensemble of regression trees (in this study, 500) where observations are repeatedly partitioned based on the ‘best’ individual split. This split point is indicative of a measure of importance reflecting the magnitude of change in abundance. The predictive power of individual RF models (R^2^f) is explained by the proportion of out-of-bag variance for each species [60] and the importance of each predictor variable (R^2^; a dimensionless value representing cumulative importance). Model performance degradation was used to select variables included in the final model as each environmental predictor is randomly permuted [63]. Multicollinearity between predictor variables is accounted for by using a conditional approach, allowing RF models to be robust to highly correlated variables.

GF modeling aggregates split importance values across each environmental gradient that were determined by the RF models, where species models with positive fits (R^2^f > 0) are collated to form distributions reflecting compositional turnover relative to each environmental predictor [60,63]. As the distribution is formed, individual RF models with higher predictive importance (i.e., high R^2^f) have a greater influence on the turnover distribution than models with lower predictive importance (i.e., low R^2^f). The shape of the distribution constructed for each environmental variable indicates the predicted rate of compositional change along the respective gradient, where increased slope steepness indicates an increased rate of community compositional turnover [60,63]. Each GF model was bootstrapped 100 times to gauge model performance and certainty. In each bootstrap iteration, a random subsample of the macroinvertebrate data was taken, and each measure of compositional turnover was integrated when constructing final GF models for each environmental predictor. All GF analyses were conducted in statistical software R version 4.1.0 [64].

#### 2.6.2. Benthic Macrofauna–Defining Tidal Zones

To examine if there were distinct shifts in macrofauna community structures with water column depth and the other environmental variables, a hierarchical cluster analysis with the SIMPROF test [65] was performed on square-root transformed macrofauna abundance data. We aimed to reveal if there were unidentified assemblages of sites that could group together based on community and environmental similarities, which would enable a closer examination of community changes with SLR (i.e., intertidal areas becoming subtidal). Three groups of clusters were essentially identified based on the macrofauna community structure, and together with the environmental data, they broadly represent different tidal zones (Supplementary Figure S1). The (dis)similarities in community structure between clusters were assessed with a similarity percentage analysis SIMPER; [65]. In order to ensure adequate sampling effort within the identified clusters, species accumulation curves (SAC) were produced by plotting the number of species against the number of sites surveyed (Supplementary Figure S2).

A distance-based redundancy analysis (dbRDA) ordination plot was used to illustrate the relationship between the set of the most influential environmental predictors (represented as vector overlays that indicate direction and strength), explaining the disparities in community structure. Collinearity between predictors was examined, but no action was required (all r < |0.8|). The multivariate analyses were conducted in PRIMER version 7.0.13 [65].

#### 2.6.3. Functional Group Analysis–Implications for Ecosystem Functioning

In order to investigate potential implications for ecosystem functioning associated with environmental conditions, shifts in macrofauna community structure and associated functional groups with SLR were examined. To confirm if the functional group community structure also differed between the initial cluster groups, a one-way PERMANOVA and PERMDISP were employed together with post-hoc pairwise tests using the functional group abundance data (square-root transformed). SIMPER analysis was performed using Bray-Curtis dissimilarities to identify the contributions of each functional group to the overall dissimilarity between the clustered groups. The analyses were conducted in PRIMER 7 with the PERMANOVA+ add-on [66].

## 3. Results

### 3.1. Relative Importance of Environmental Gradients for Predicting Compositional Turnover

Gradient Forest (GF) analysis was employed to investigate thresholds of community compositional turnover for environmental gradients known to influence community structure. GF effectively modeled taxa turnover for 85 of the 157 input taxa based on 100 bootstrapped model runs. All 12 environmental predictor variables included were considered important for predicting patterns of macroinvertebrate community compositional turnover, contributing to 48% combined cumulative importance. Depth was, however, revealed as the most important predictor (6.6% of the conditional importance), followed by sediment Chl *a* concentration (6.5% of the conditional importance). Other environmental gradients considered important predictors by GF were average current speed, gravel, copper (Cu), mud content, total phosphorous (TP), lead (Pb), total nitrogen (TN), organic content (OM), zinc (Zn) and sand content (3–5% of the conditional importance each).

**Figure 2:**
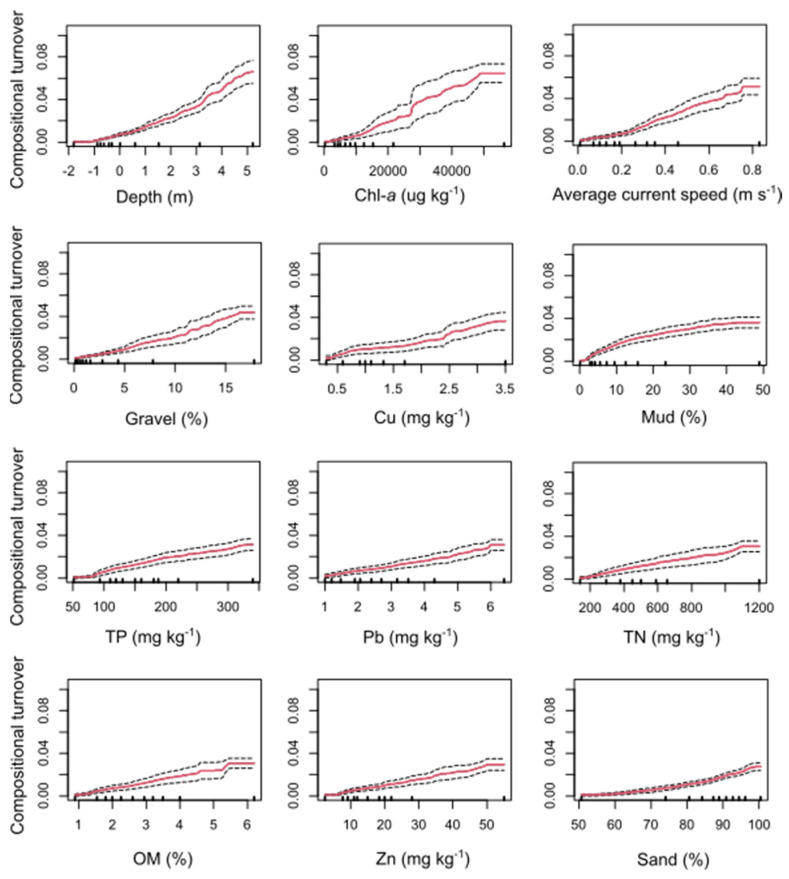
Cumulative importance curves (with 95% prediction intervals) visualizing the overall pattern of compositional turnover (in R^2^-importance units) for all species across all environmental predictors included in gradient forest models. Rug plots along the x-axis represent deciles across each environmental gradient.

Non-linear curves representing rates of macroinvertebrate compositional turnover were observed for all environmental gradients except for Pb and sand, which had comparatively linear relationships indicating a constant rate of compositional turnover for these predictors (Figure 2). Steep sections in the cumulative importance curves indicated large shifts in community structure (i.e., rapid compositional turnover), whereas plateaued sections of the curves indicated more comparable communities. For depth, relatively constant rates of compositional turnover were observed but with a few rapid changes around 1, 3, and 4.5 m (Figure 2). For Chl *a*, the turnover rates increased relatively constantly, but around 30,000 µg/kg, a rapid increase was indicated. However, the variability in mean predicted cumulative change (measured by the 95% prediction interval) was noticeably high due to few data points above this value. The compositional turnover along the gradient of average current speed indicated gradual rates of increase at low current speeds but larger change around 0.3, 0.5, and 0.7 m/s. Regarding grain size, there were low turnover rates until 3% mud content, followed by more rapid changes. Similar patterns were indicated for the nutrients (TP and TN), low turnover with low nutrient concentrations followed by steadily increasing turnover.

### 3.2. Definition of Tidal Zones Based on Community and Environmental Data

**Figure 3:**
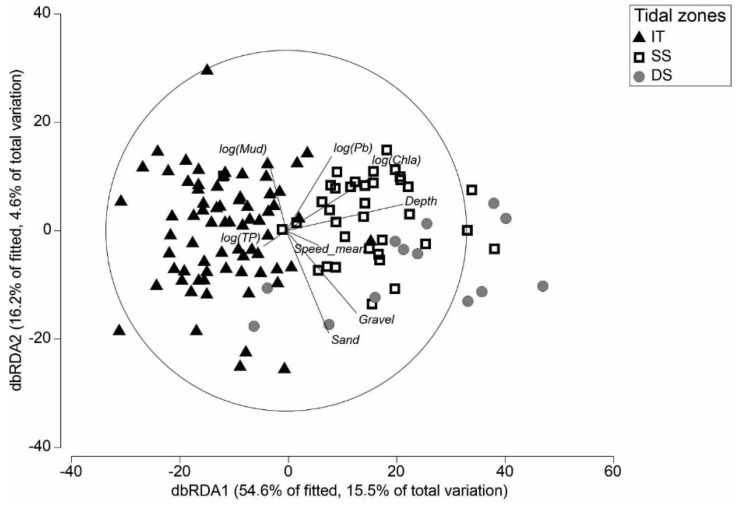
Distance-based redundancy analysis (dbRDA) plot visualizing the direction and influence of environmental predictors on shifts in macroinvertebrate community structure.

Hierarchical cluster analysis performed on the complete species abundance dataset indicated six macrofaunal community clusters on a level of 37% similarity (Supplementary Figure S1). Based on a comparison of the inter-cluster characteristics, similar sites were combined into clusters representing three tidal zones (intertidal IT, shallow subtidal SS, and deep subtidal DS) for further analysis. The IT sites were shallowest, with an average water depth of −0.6 m (Table 2), compared to the SS and DS sites, with average depths of 1.5 m and 3.0 m, respectively. There was a small number of sites that overlapped in depth between groups. However, the average depths, environmental characteristics, and position within the harbor (Table 2, Figure 1) indicated that these clusters represented different tidal zones. All sites were generally sandy (>85% sand on average), but the mud and OM content varied as expected between the tidal zones, with the highest values at IT sites compared to SS and DS sites (Table 2). The average current speeds were accordingly lowest at the IT (0.15 m/s) sites, compared to SS (0.33 m/s) and DS (0.53 m/s) sites. The environmental variables explaining the variation in the macrofauna community composition at each site were illustrated by a dbRDA (Figure 3). Separation of the tidal zones occurred along the x-axis, aligning with the variables depth, chlorophyll *a* and average current speed, and along the y-axis due to sediment characteristics, mud, sand, and gravel content.

### 3.3. Differences in Macrofauna Communities between Tidal Zones

Species accumulation curves (SAC) were generated to provide evidence that IT, SS, and DS clusters had been sampled adequately. For IT and SS sites, the SAC indicated that adequate sampling (i.e., a noticeable decrease in species accumulation rates with increasing sampling effort) occurred after 10 sites (30 cores) and 12 sites (36 cores), respectively (Supplementary Figure S2). For the DS cluster, species accumulation rates were less clear due to fewer samples, and results regarding this group need to be interpreted with care.

In this study, there were 157 different taxa identified across all sites. The highest number of taxa occurred in the SS tidal zone, with a total of 126 taxa recorded, compared to the IT and DS tidal zone, with a total of 83 and 66 taxa recorded, respectively. The abundance per core was also highest in the SS (234 ind./core) compared to the IT (109 ind./core) and DS (70 ind. per core; Table 2). Shannon-Wiener diversity indices for all three tidal zones were similarly varying between the tidal zones, with the highest at SS, then IT, and the lowest at DS (2.02, 1.92, and 1.72).

Of the top five most abundant taxa determined for each zone, IT had three taxa in common with SS (Amphipoda, Spionidae, and *Heteromastus filiformis*) and only one taxon (Amphipoda) shared with DS (Table 2). Similarly, there was only one taxon in common among the SS and DS top five most abundant (Amphipoda). A SIMPER analysis revealed that overall dissimilarity between IT and SS sites was 66%, and this was largely driven by differences in taxa abundance of Amphipoda (e.g., Caprellidae), Spionidae polychaetes (e.g., *Aonides trifida*, *Boccardia syrtis*), the polychaete *Aricidea* sp., oligochaete worms and the polychaete *Heteromastus filiformis* (Supplementary Table S3). The overall dissimilarity between IT and DS was 77%, where differences in community structure was primarily attributed to the polychaetes Spionidae (e.g., *A. trifida*, *B. syrtis*), Amphipoda (e.g., Caprellidae), the bivalve *Paphies australis* and polychaete *H. filiformis* (Supplementary Table S3). There was 72% dissimilarity between SS and DS community structures, largely attributed to Spionidae polychaetes (e.g., *A. trifida*, *B. syrtis*), oligochaete worms, polychaetes *H. filiformis*, *Aricidea* sp., Amphipoda (e.g., Caprellidae) and bivalve *P. australis*. The top taxa (except *P. australis*) generally had greater abundances at the IT and SS sites than at the DS sites (Supplementary Table S3).

### 3.4. Functional Group Analysis

The functional group structures between the different tidal zones were analysed to reveal if the shift in macrofauna communities potentially translates into a shift in the functionality of the benthic ecosystems. Significant differences in functional group structure between all tidal zones were indicated (PERMANOVA; Pseudo-F = 15.09; *p* < 0.001; Supplementary Table S4). Additionally, there were homogenous dispersions (PERMDISP *p* > 0.05) between the tidal zones except between SS and DS tidal zones (PERMDISP *p* < 0.05). Subsequently, the functional group differences between SS and DS should be interpreted with care.

A SIMPER analysis revealed that overall dissimilarity between the IT and SS functional group communities was 51% and was largely driven by differences in functional group abundance of FG13 (Soft-bodied, deposit-feeding, below the surface, limited mobility; e.g., polychaete *H. filiformis*), FG22 (Rigid, deposit-feeding, predator/scavenger, top 2 cm, mobile; e.g., Amphipoda), FG12 (Soft-bodied, deposit-feeding, below the surface, mobile; e.g., polychaete Spionidae), FG17 (Soft-bodied, predator/scavenger, top 2 cm, limited mobility; e.g., polychaetes Syllidae), FG19 (Soft-bodied predator/scavenger, below the surface, limited mobility; e.g., Oligochaeta) (Supplementary Table S5). In most instances, functional group abundance tended to be lower in IT compared to SS, with the exception of FG2 (Calcified, suspension-feeding, top 2 cm, mobile; e.g., bivalve *A. stutchburyi*) and FG6 (Calcified, deposit-feeding, top 2 cm, limited mobility; e.g., bivalve *L. hartvigiana*), where average abundance per core was greater for IT than SS (by factors of 1.8 and 2.8 respectively). The overall dissimilarity between IT and DS was 58%, mostly attributed to abundance differences of FG12 (Soft-bodied, deposit-feeding, below surface, mobile; e.g., polychaetes Spionidae), FG22 (Rigid, deposit-feeding, predator/scavenger, top 2cm, mobile; e.g., Amphipoda), FG2 (Calcified, suspension-feeding, top 2 cm, mobile; e.g., bivalve *A. stutchburyi*), FG13 (Soft-bodied, deposit-feeding, below the surface, limited mobility; e.g., polychaete *H. filiformis*), FG19 (Soft-bodied, predator/scavenger, below surface, limited mobility; e.g., Oligochaeta) (Supplementary Table S5). There was 56% dissimilarity between SS and DS, mostly driven by differences in FG12 (Soft-bodied, deposit-feeding, below surface, mobile; e.g., polychaete Spionidae), FG13 (Soft-bodied, deposit-feeding, below the surface, limited mobility; e.g., polychaete *H. filiformis*), FG22 (Rigid, deposit-feeding, predator/scavenger, top 2cm, mobile; e.g., Amphipoda), FG19 (Soft-bodied, predator/scavenger, below the surface, limited mobility; e.g., Oligochaeta), FG2 (Calcified, suspension-feeding, top 2 cm, mobile; e.g., bivalve *A. stutchburyi*).

## 4. Discussion

The aim of this study was to attempt to fill gaps in the scientific literature around the implications of sea level rise (SLR) on estuarine biodiversity and ecosystem functioning. To date, there has been little research addressing this aspect of coastal climate change ecology despite the growing relevance of diffuse climate change stressors. The findings indicated that there would be significant shifts in estuarine macroinvertebrate community structure with future SLR. Additionally, some species-specific shifts may trigger functional consequences. For example, the functionally important large cockle *Austrovenus stutchburyi*, in the intertidal zone, is unlikely to have a substitute in the shallow subtidal zone. The results thus demonstrate that localized gains and losses of individual species and functional traits within the community will likely have implications for the overall estuarine ecosystem functioning.

### 4.1. Environmental Drivers of Macroinvertebrate Community Structure and Compositional Turnover

Water column depth was identified as the most important predictor of rates of community compositional turnover. The influence of depth on the spatial distribution of marine organisms has been well studied, e.g., [67,68,69,70,71]. However, links to SLR are generally ignored. In estuaries, increasing depths will be a key outcome of SLR [72]. Therefore, gaining an understanding of how macroinvertebrate communities shift with depth allows us to consider the prospective implications of SLR.

The GF modeling indicated constantly increasing compositional turnover rates of macroinvertebrate communities with increasing depth and rapid changes around 1, 3, and 4.5 m. Using SLR predictions with current global emission rates, we can expect a rise between 0.6 to 1.1 m by 2100 [25], and regional predictions based on different climate change scenarios, including local variations in the harbor estimate a range of 0.3–1 m [50]. Using depth as a proxy for SLR (assuming spatial and temporal variability is equal; [44]), the results indicated that the upper prediction reflects a threshold where a small increase in SLR at 1 m will drive a disproportionately greater change in macroinvertebrate community structure than that perceived for preceding SLR scenarios. This may be explained by the expected reductions suffered by intertidal species that are constrained by their optimal spatial distribution [73,74,75], impeding their ability to thrive in deeper submerged habitats. This shift observed in macroinvertebrate community structure would also align with that expected of the projected intertidal habitat loss under a 1.1 m SLR scenario (~85% reduction by the year 2100; [18]). Nonetheless, steady rates of compositional turnover were still observed approaching 1 m depth, providing an indication that even small changes in SLR will alter macroinvertebrate community structure, perhaps irreversibly, within Tauranga Harbor, as also earlier indicated by modeled distributions for a subset of species by Rullens and Mangan et al. [20].

These findings highlight the importance of depth as a predictor of species and community responses to SLR. However, it is unlikely that depth alone is driving observed responses, but instead acts as a surrogate for a combination of co-varying factors known to shape patterns of estuarine macroinvertebrate biodiversity. The high relative importance of depth may be owed to relationships with water column and sedimentary environment characteristics such as sea temperature, salinity, sediment grain size, and nutrient content, which are all known to also influence patterns of macroinvertebrate biodiversity [76,77,78,79,80]. This suggests that depth can represent a host of co-varying environmental parameters that will also shift with SLR.

The gradient forest analysis also indicated average current speed as an important factor for predicting patterns in macroinvertebrate community structure and compositional turnover (Figure 2). Flow rates are often highly variable throughout estuaries, largely owed to the complex bathymetry of the seafloor (e.g., channels) and bordering landforms (e.g., tombolos) that influence flow dynamics [81,82]. Here, average current speeds measured at each site varied from 0.01–0.83 m/s (Table 2), with a rapid increase in compositional turnover rate around current speeds of 0.3, 0.5, and 0.7 m/s (Figure 2). Average current speeds exceeding this value generally existed at deeper sites in the harbor, often in the center of channels, which is likely explained by the strong influence of tidal exchange on current speeds in main channels [83,84].

The flow dynamics associated with these channels often support increased delivery rates of particulate food, which is favorable to filter-feeding organisms [85]. This may explain why high densities of the filter-feeding bivalve *Paphies australis* were generally restricted to deep subtidal sites in this study, as also shown in earlier studies e.g., [73]. As estuary depth is expected to increase with future SLR, we can also anticipate altered current speeds (i.e., likely reduced in deep channels) due to the influence of basin geometry (e.g., degree of channel constriction) and depth on flow dynamics [56,86]. This indicates that although overall water column depth will increase with SLR, which could suggest that species like, for example, *P. australis* will extend their spatial distribution, their distribution is likely to be constrained if altered current speeds do not match those required to support high densities. From this, we can deduce that some species will not necessarily extend their spatial distribution to ‘follow’ their optimal depth range if other environmental factors are altered that may limit their distribution.

### 4.2. Comparisons across Tidal Zones and Implications of Reduced Intertidal Area

Estuarine benthic macroinvertebrate community structure differed across intertidal (IT), shallow subtidal (SS), and deep subtidal (DS) zones. The findings demonstrated that species richness and average abundance were highest at the SS sites (Table 2). An explanation for this is that SS represents a transitional zone comprising a mixture of species that occur in IT and DS habitats [87,88]. The lower species richness and average abundance at the IT sites were expected as many estuarine species lack unique adaptations (e.g., desiccation prevention) required to endure environmental circumstances typical of IT habitats, (e.g., air exposure during periods of tidal emergence) [89]. Thus, the subtidal habitat is preferable to more species. Although the general consensus within ecological studies is that increased diversity positively influences ecosystem function [90,91,92], this can be context dependent [93,94]. In estuarine ecosystems, certain species make a disproportionate contribution to ecosystem function (e.g., *Austrovenus stutchburyi*) [36] owed to key factors (e.g., abundance/dominance, functional traits) influencing important ecological processes and functions (e.g., sediment destabilization, primary production, ecosystem engineering) [30,34,35,41,95,96]. It is thus critical to recognize that greater species richness does not always reflect better ecosystem performance, particularly when functionally important or unique species are reduced or lost [94].

Under future SLR conditions, it is suggested that intertidal areas will essentially become subtidal as they become permanently inundated [72]. In this study, functional group community structure significantly differed between IT, SS, and DS habitats, indicating dominant IT functional groups may experience reductions whilst those of SS will become more widespread. Based on our results, such a shift would suggest a two-fold increase in the average abundance of soft-bodied deposit-feeders located below the sediment surface, such as polychaetes (FG13 and FG12), in areas where this habitat shift occurs. Species included in this functional group are considered important drivers of community structure, and many are key bioturbators that contribute to ecological processes and promote ecosystem function (e.g., nutrient cycling, sediment destabilization) [97,98], which at a glance suggests this shift could be desirable. Additionally, these functional groups were relatively abundant across all tidal zones and had a high degree of redundancy, indicating high ecological resilience to environmental change. However, the results also suggested that calcified suspension- and deposit-feeders at the sediment surface, such as the bivalves *Austrovenus stutchburyi* and *Linucula hartvigiana* (FG2 and FG6), will experience reductions to less than half of the average abundance in areas that shift from IT to SS, indicating this shift may have a large impact on the ecosystem. The dominant species of FG2, *A. stutchburyi*, plays an important role in intertidal habitats as an ecological engineer and positively influences ecological processes such as primary production, denitrification, and reworking of the sedimentary environment [31,36,99,100]. Additionally, the distribution of species contributing to these dominant IT functional groups is generally more spatially constrained and displays lower redundancy. Therefore the expected habitat shifts associated with SLR indicate implications for ecosystem function due to the predicted reductions suffered by these groups.

There is often a degree of functional redundancy within estuarine taxa where multiple species can offer similar contributions to ecosystem processes [59]. However, *A. stutchburyi* and *Paphies australis* were the only abundant species characterized by FG2 (suspension-feeding, mobile, top 2 cm), indicating low redundancy and resilience despite occurring in high abundances. Furthermore, as *P. australis* is generally restricted to subtidal regions, particularly where depth and current speeds are greater [73] (i.e., DS habitats in this study), it is unlikely to move into all newly submerged areas due to SLR (equivalent to SS habitats in this study). Thus, despite similar contributions to functionality, *P. australis* is unlikely to provide functional resilience should intertidal *A. stutchburyi* populations be reduced or lost following a shift to shallow subtidal habitats. This is concerning as SLR will reduce intertidal area [8,18,101], whilst shallow subtidal coverage is expected to increase. Furthermore, modeling studies in this estuary have indicated that SLR will cause the loss of high-density areas of *A. stutchburyi* and these locations coincide with areas that exhibit the highest potential for ecosystem services [20,102]. This highlights the vulnerability of *A. stutchburyi* and its functional role in intertidal habitat loss. Thus, we can anticipate significant implications on ecosystem functions and the ecosystem services they underpin due to SLR.

An important aspect to acknowledge is the possibility that time scales associated with geomorphic and ecological shifts due to SLR may differ. Generally, ecological shifts can occur very rapidly as changing environmental conditions can often have a direct impact on species distributions [103]. Changes to geomorphology, however, can take place over a much longer period of time [104]. This suggests that if intertidal habitats become flooded by SLR, the projected changes to the sedimentary environment (i.e., lower mud content/coarser sediments) may display a time lag, whereas the response of species distributions and their respective communities to SLR is expected to be much more immediate. Moreover, there is the possibility of intertidal habitat ‘legacy effects’ (i.e., residual qualities of the former habitat) [105] hindering the transition of ‘muddy’ sediments to ‘sandy’ sediments typical of the shallow subtidal habitats observed in this study. This suggests that species currently thriving in shallow subtidal habitats (e.g., some polychaetes) may not necessarily occur in the same densities in inundated intertidal areas if sediment composition limits their distribution. As we employed a space-for-time approach, key findings of this study heavily rely on the assumption that intertidal habitats will essentially become shallow subtidal as they become permanently inundated by SLR. Therefore, unknown legacy effects of intertidal habitats may influence macroinvertebrate community responses to SLR that have not been accounted for in this study.

## 5. Conclusions

SLR resulting from a warming planet will significantly modify coastal geomorphology, influencing tidal dynamics, currents, and the sedimentary environment. Reduced intertidal coverage will impact estuarine ecosystems and their communities at local and global scales, yet the ecological repercussions have been largely dismissed despite their prospective significance. This study demonstrated that macrofauna community structure differed significantly across tidal zones and that patterns in macrofauna biodiversity will shift in response to altered depth and concomitant changes to the water column and sedimentary environment. Thus, it highlights that SLR will significantly alter estuarine macroinvertebrate communities and subsequently result in repercussions for ecosystem function and resilience. The results of this study also indicated that the ecological impacts of species loss would be dependent on the species-specific contributions to ecosystem function. Many species-specific contributions are, however, unknown, which may mean that there are implications that are not yet fully recognized for ecosystem function if intertidal habitats are lost to SLR. We do, however, know that intertidal habitats hold a significant role in maintaining important ecological processes (e.g., primary production, and denitrification) [106,107], often exceeding that of the adjacent subtidal habitats [108]. Additionally, species abundance has been shown to strongly influence these processes, e.g., [32,109]. Therefore, it is reasonable to assume that highly abundant intertidal species will have a significant influence on ecological processes, although the nature of the effect will likely depend on species identity. To obtain an extensive understanding of the implications, we can expect to arise under future SLR conditions. We must understand the unique roles of all species and their functional roles that are vulnerable to expected habitat shifts. Management efforts targeting biodiversity in coastal environments should also recognize the expected shifts in community structure that will occur through habitat loss. This will be fundamental for ensuring management strategies are indeed effective for maintaining biodiversity, particularly for systems such as marine protected areas that often treat habitats as fixed in space over time. Well-informed management of biological communities and coastal environments will be critical for ensuring that the ecosystem functions and services valued by society are conserved for future generations.

## Figures and Tables

**Table 1:** Summary of defining traits for each functional group (1–26) as described by Greenfield et al. [59]. An example species are given for each group. Taxonomic class indicated within brackets; T = Thecostraca, B = Bivalvia, G = Gastropoda, A = Anthozoa, P = Polychaeta, M = Malacostraca.

Functional Group	Description of Traits	Example Species
1	Calcified, Suspension feeding, Attached	*Austrominius modestus* (T)
2	Calcified, Suspension feeding, Top 2 cm, Freely mobile	*Austrovenus stutchburyi* (B)
3	Calcified, Suspension feeding, Top 2 cm, Limited mobility	*Arthritica bifurca* (B)
4	Calcified, Suspension feeding, Top 2 cm, Sedentary	*Arcuatula senhousia* (B)
5	Calcified, Deposit/Pred.Scav/Grazer, Above surface, Freely mobile	*Zeacumantus subcarinatus* (G)
6	Calcified, Deposit feeding, Top 2 cm, Limited mobility	*Linucula hartvigiana* (B)
7	Calcified, Deposit feeding, Predator/Scavenger, Top 2 cm, Freely mobile	*Pisinna zosterophila* (G)
8	Calcified, Deposit feeding, Deep, Limited mobility, No habitat structure, Large	*Macomona Liliana* (B)
9	Soft-bodied, Suspension feeding, Attached	*Anthopleura aureoradiata* (A)
10	Soft-bodied, Suspension feeding, Tube structure	*Euchone* sp. (P)
11	Soft-bodied, Deposit feeding, Top 2 cm, Freely mobile	Spaerodoridae (P)
12	Soft-bodied, Deposit feeding, Below surface, Freely mobile	Spionidae (P)
13	Soft-bodied, Deposit feeding, Below surface, Limited mobility	*Heteromastus filiformis* (P)
14	Soft-bodied, Deposit feeding, Deep	*Hyboscolex longiseta* (P)
15	Soft-bodied, Below surface, Tube structure	Terebellidae (P)
16	Soft-bodied, Predator/Scavenger, Top 2 cm, Freely mobile	Sigalionidae (P)
17	Soft-bodied, Predator/Scavenger, Top 2 cm, Limited mobility	Syllidae (P)
18	Soft-bodied, Predator/Scavenger, Below surface + Deep, Freely mobile, No habitat structure	*Perinereis* sp. (P)
19	Soft-bodied, Predator/Scavenger, Below surface, Limited mobility	Oligochaeta
20	Soft-bodied, Above surface, Top 2 cm, Below surface, Deep, Sedentary, Tube structure	*Owenia petersenae* (P)
21	Rigid, Suspension feeding, Top 2 cm	Tanaidacea (M)
22	Rigid, Deposit feeding, Predator/Scavenger, Top 2 cm, Freely mobile, No habitat structure	Amphipoda (M)
23	Rigid, Above surface, Freely mobile	Cumacea (M)
24	Rigid, Above surface, Freely mobile, Large	Ophiuroidea
25	Rigid, Predator/Scavenger, Attached	No individuals identified
26	Rigid, Predator/Scavenger, Below surface, Freely mobile, Large burrow former	*Hemiplax hirtipes* (M)

**Table 2:** Summary of average environmental and univariate macrofauna diversity measures (min-max) measured in Tauranga Harbor as a function of tidal zones; intertidal (IT, n = 70), shallow subtidal (SS, n = 36) and deep subtidal (DS, n = 13).

		IT	SS	DS
**Environmental Variables**				
	Depth (m)	−0.6 (−2.0–3.0)	1.5 (−1.0–7.9)	3.0 (−0.2–9.0)
	Mud (%)	13.6 (0.1–76.4)	9.0 (2.6–25.4)	3.0 (0.6–5.0)
	Sand (%)	85 (24–100)	87 (67–96)	91 (78–99)
	Gravel (%)	1.8 (0.1–14.6)	4.7 (0.1–15.0)	5.9 (0.1–17.8)
	OM (%)	2.9 (0.9–10.0)	2.8 (1.3–6.2)	1.7 (1.0–3.0)
	Chl *a* (µg/kg)	6107 (210–16,000)	16,678 (5900–41,300)	17,685 (2000–56,300)
	TP (mg/kg)	168 (51–580)	152 (79–340)	121 (81–180)
	TN (mg/kg)	484 (140–1900)	548 (499–1200)	452 (190–499)
	Cu (mg/kg)	1.3 (1.0–6.1)	1.1 (0.4–3.5)	0.7 (0.3–1.0)
	Pb (mg/kg)	2.7 (1.0–13.0)	3.0 (1.6–6.4)	2.0 (1.0–3.8)
	Zn (mg/kg)	17.7 (2.5–55.0)	17.7 (7.7–37.0)	12.2 (6.4–25.0)
	Av. current speed (m/s)	0.15 (0.01–0.52)	0.33 (0.01–0.67)	0.53 (0.23–0.83)
**Benthic community**				
	S (taxa per core)	19 (6–31)	25 (18–37)	15 (10–21)
	N (ind. per core)	109 (27–329)	234 (49–744)	70 (22–183)
	Occurrence (% of sites taxa occurs at)	23 (1–100)	20 (3–100)	23 (8–100)
	H’ (per core)	1.92 (0.11–2.71)	2.02 (0.76–2.74)	1.72 (0.45–2.55)
	Most abundant taxa	Amphipoda (M) Spionidae (P) *Heteromastus filiformis* (P) *Austrovenus stutchburyi* (B) *Linucula hartvigiana* (B)	Spionidae (P) Amphipoda (M) Oligochaeta *Aricidea* sp. (P) *Heteromastus filiformis* (P)	*Paphies australis* (B) Amphipoda (M) Hesionidae (P) Syllidae (P) *Magelona* sp. (P)

Chl *a* Chlorophyll *a*, TN total nitrogen, TP total phosphorus, Cu Copper, Pb Lead, Zn Zinc, OM organic content, Av. average current speed, S average number of taxa, N average abundance, H’ Shannon-Wiener diversity index. Class indicated for the most abundant taxa: M Malacostraca, P Polychaeta, B Bivalvia.

## Data Availability

Restrictions apply to the availability of these data. Data was obtained from the Research programs Manaaki Taha Moana (MTM) and Oranga Taiao Oranga Tangata (OTOT) and can be made available upon request to Dana Clark (Cawthron Institute) with the permission of the Research Programmes.

## Supplementary Materials

The following supporting information can be downloaded at: https://www.mdpi.com/article/10.3390/biology12010105/s1, Table S1: Environmental and biological variables at each site; Table S2: Summary of analysis methods and units of the measured environmental variables; Figure S1: Cluster analysis of the benthic communities for identification of tidal zones; Figure S2: Species accumulation curves for each tidal zone; Table S3: SIMPER analysis results based on species abundance data within tidal zones; Table S4: Results of PERMANOVA comparing functional group composition between tidal zones; Table S5: SIMPER analysis results based on functional group data within tidal zones.
