# Supplementary material for: Estimating Effects of Sea Level Rise on Benthic Biodiversity and Ecosystem Functioning in a Large Meso-Tidal Coastal Lagoon
Source: Biology (Basel). 2023 Jan 10;12(1):105. doi: 10.3390/biology12010105 (PMC9855350; doi:10.3390/biology12010105)
Supplement: Supplementary file 1 [file biology-12-00105-s001.zip › biology-2109154-supplementary.pdf]

SUPPLEMENTARY MATERIAL

for

Estimating effects of sea-level rise on benthic biodiversity and ecosystem functioning in a large meso-tidal coastal lagoon

by

Olivia Dixon, Johanna Gammal, Dana Clark, Joanne Ellis, Conrad A. Pilditch

Supplementary Table S1. Site information. Data were obtained then collated from the research programmes MTM (Ellis et al. 2013 [51]; Site ID I1-I75, sampled during austral summer Dec 2011–Feb 2012) and OTOT (Clark et al. 2018 [52]; Site ID S1-S45, sampled during austral autumn Mar–May 2016). Tidal zone refers to the tidal zones derived from the clustered groups (IT: intertidal, SS: shallow subtidal, DS: deep subtidal), depth is the model grid depth corrected to chart datum (negative values reflect periods of emergence).

**Site ID**	**NZTM E**	**NZTM N**	**Tidal zone**	**Depth**	**Average current speed**	**Chl-*a***	**OM**	**Mud**	**Sand**	**Gravel**	**Pb**	**Zn**	**Cu**	**TP**	**TN**	**N**	**S**
				m	m/s	µg/kg	%	% <63 µm	% ≥63µm <2mm	% ≥2 mm	mg/kg	mg/kg	mg/kg	mg/kg	mg/kg	ind./core	no./core
I1	1862844	5850790	IT	-0.84	0.14	4600	1.6	3.6	96.0	0.4	1.1	8	1	110	380	107 ± 18	18 ± 2
I2	1863039	5849578	IT	0.27	0.33	6600	2.4	2.5	87.3	10.2	1.4	9	1	210	590	236 ± 49	16 ± 3
I3	1861313	5849572	IT	-0.87	0.12	4600	1.9	3.9	94.5	1.7	1.0	6	1	110	380	145 ± 61	15 ± 2
I4	1860898	5848973	IT	-1.12	0.14	3200	2.5	5.6	94.0	0.4	1.0	6	1	120	350	108 ± 39	12 ± 2
I5	1862374	5849061	IT	-0.32	0.28	2400	1.6	1.5	97.4	1.2	1.0	7	1	140	290	256 ± 109	15 ± 4
I6	1860947	5847735	IT	-0.68	0.17	8600	3.8	7.3	91.9	0.7	1.3	11	1	180	530	120 ± 24	18 ± 1
I7	1860284	5846213	IT	-0.91	0.15	10000	3.0	10.2	88.7	1.1	1.0	11	1	180	640	195 ± 45	15 ± 2
I8	1862990	5845258	DS	0.39	0.47	5300	3.0	2.9	96.7	0.3	1.3	10	1	160	380	46 ± 21	6 ± 1
I9	1861385	5842837	IT	-0.71	0.09	2200	1.0	3.6	94.9	1.5	1.4	27	1	78	180	95 ± 12	12 ± 2

Site ID	NZTM E	NZTM N	Tidal zone	Depth	Average current speed	Chl-*a*	OM	Mud	Sand	Gravel	Pb	Zn	Cu	TP	TN	N	S
				m	m/s	µg/kg	%	% <63 µm	% ≥63µm <2mm	% ≥2 mm	mg/kg	mg/kg	mg/kg	mg/kg	mg/kg	ind./core	no./core
I10	1859160	5841668	IT	-0.78	0.10	1100	4.4	30.9	66.2	2.9	5.6	34	3	340	1000	104 ± 79	9 ± 1
I11	1861019	5840122	IT	-0.60	0.12	4400	2.8	6.5	92.5	0.9	1.1	12	1	120	390	152 ± 87	12 ± 2
I12	1860872	5838709	IT	-0.55	0.23	1900	2.0	8.9	91.0	0.1	1.9	9	1	120	300	118 ± 39	11 ± 2
I13	1859645	5838849	IT	-0.97	0.10	2800	3.1	14.3	85.1	0.6	3.4	22	1.5	250	540	34 ± 17	8 ± 2
I14	1858852	5837443	IT	-1.05	0.08	5600	4.5	24.7	74.3	1.0	4.6	26	2.4	330	830	119 ± 49	9 ± 2
I15	1864615	5844595	IT	1.11	0.42	1200	2.1	3.7	95.9	0.4	2.2	13	1	160	340	160 ± 89	17 ± 4
I16	1861664	5847064	IT	0.46	0.27	7000	1.8	3.3	96.1	0.7	1.5	11	1	180	310	236 ± 35	19 ± 2
I17	1866194	5839926	IT	-0.29	0.19	4200	2.1	3.9	94.1	2.0	1.0	8	1	110	370	111 ± 49	15 ± 3
I18	1868133	5838623	IT	-1.26	0.15	210	0.9	1.3	98.5	0.2	1.1	3	1	53	140	38 ± 4	11 ± 2
I19	1865401	5839681	IT	-0.07	0.24	3000	2.1	4.3	95.6	0.1	1.2	7	1	91	310	27 ± 7	10 ± 2
I20	1870400	5836995	IT	-1.30	0.18	1200	1.6	0.1	100	0.1	1.3	10	1	92	340	34 ± 4	9 ± 0
I21	1862901	5839911	IT	-0.58	0.16	5100	3.8	6.5	91.3	2.0	1.3	11	1	180	540	98 ± 31	15 ± 6
I22	1864304	5836733	IT	-0.51	0.19	3300	4.2	17.5	81.6	0.9	3.5	18	1.7	220	700	82 ± 16	11 ± 3
I23	1863958	5837013	IT	-1.02	0.12	7900	3.1	34.2	64.9	0.8	3.1	14	1	200	430	81 ± 41	11 ± 1
I24	1867485	5834361	IT	-0.17	0.22	5600	2.6	15.7	83.5	0.8	3.1	19	1	200	390	97 ± 22	13 ± 2
I25	1867748	5834506	IT	-0.86	0.07	3800	0.9	1.4	97.2	1.4	1.4	6	1	51	180	43 ± 17	9 ± 1

Site ID	NZTM E	NZTM N	Tidal zone	Depth	Average current speed	Chl-*a*	OM	Mud	Sand	Gravel	Pb	Zn	Cu	TP	TN	N	S
				m	m/s	µg/kg	%	% <63 µm	% ≥63µm <2mm	% ≥2 mm	mg/kg	mg/kg	mg/kg	mg/kg	mg/kg	ind./core	no./core
I26	1864309	5834506	IT	-0.81	0.13	3600	4.0	23.3	76.5	0.1	2.7	13	1.3	130	590	51 ± 21	13 ± 2
I27	1863615	5834467	IT	-0.52	0.17	7300	4.2	18.7	81.3	0.1	4.3	20	2.2	180	580	75 ± 44	7 ± 2
I28	1862821	5834277	IT	-0.95	0.08	8600	3.5	22.4	77.5	0.1	2.8	14	1.3	160	520	329 ± 171	3 ± 1
I29	1872451	5833584	IT	-0.59	0.12	3900	2.7	16.2	82.6	1.2	2.5	16	1.2	150	690	76 ± 2	14 ± 2
I30	1872580	5833532	IT	-0.48	0.11	4000	1.8	8.9	90.7	0.5	1.9	9	1	97	450	90 ± 26	14 ± 2
I31	1875571	5831455	IT	-0.84	0.16	4800	3.2	13.0	86.1	1.0	1.9	10	1	120	490	69 ± 31	15 ± 2
I32	1875642	5830632	IT	-0.57	0.15	8100	2.3	7.7	91.2	1.1	2.5	12	1	160	490	113 ± 20	17 ± 1
I33	1876347	5830239	IT	-0.61	0.17	7200	2.6	6.3	91.5	2.3	2.2	12	1	190	550	112 ± 11	14 ± 3
I34	1876500	5830117	IT	-0.36	0.15	5400	1.8	3.2	96.2	0.7	1.9	8	1	130	350	101 ± 61	13 ± 3
I35	1873330	5831630	IT	-1.23	0.13	3300	1.4	3.3	96.0	0.7	1.2	5	1	93	290	70 ± 15	13 ± 2
I36	1866432	5832044	IT	1.71	0.18	4700	3.3	12.6	87.3	0.2	3.1	15	1	180	530	145 ± 18	18 ± 2
I37	1863362	5831812	IT	-0.93	0.12	3300	4.5	47.5	51.0	1.5	4.5	26	1.4	310	760	66 ± 43	9 ± 2
I38	1864247	5830677	IT	-0.63	0.07	4100	4.2	48.9	50.7	0.3	4.1	21	1.1	260	620	29 ± 10	10 ± 2
I39	1865756	5831517	IT	-0.71	0.07	4300	2.6	15.0	84.2	0.8	2.0	12	1.6	130	460	64 ± 25	14 ± 3
I40	1866557	5830113	IT	-1.32	0.02	6100	3.8	31.5	68.4	0.2	4.0	19	1.4	220	650	31 ± 2	9 ± 5
I41	1866954	5830644	IT	-0.67	0.15	5000	3.5	15.1	84.3	0.6	2.3	15	1	140	450	66 ± 16	15 ± 1

Site ID	NZTM E	NZTM N	Tidal zone	Depth	Average current speed	Chl-*a*	OM	Mud	Sand	Gravel	Pb	Zn	Cu	TP	TN	N	S
				m	m/s	µg/kg	%	% <63 µm	% ≥63µm <2mm	% ≥2 mm	mg/kg	mg/kg	mg/kg	mg/kg	mg/kg	ind./core	no./core
I42	1868018	5830518	SS	-0.17	0.17	5900	4.0	25.4	73.9	0.9	4.3	27	1.5	280	760	122 ± 37	12 ± 1
I43	1868270	5833779	IT	0.75	0.17	5000	1.6	4.9	94.5	0.6	2.6	14	1	120	310	89 ± 18	14 ± 2
I44	1870057	5830629	IT	-0.36	0.34	5000	4.3	20.9	77.6	1.6	5.1	21	1.3	220	450	184 ± 20	19 ± 3
I45	1879574	5828564	DS	0.33	0.83	11000	1.2	1.5	97.5	1.0	1.0	6	1	180	320	81 ± 21	13 ± 2
I46	1868460	5828617	IT	-0.90	0.07	4900	3.8	38.6	60.0	1.5	3.7	22	1.3	240	620	86 ± 2	14 ± 1
I47	1867687	5827666	IT	-2.00	0.01	8800	4.0	29.2	60.5	10.2	3.3	18	1	220	660	29 ± 12	8 ± 1
I48	1868434	5825385	IT	-0.97	0.04	11000	10.0	76.4	23.7	0.1	13.0	46	6.1	580	1900	148 ± 61	4 ± 1
I49	1869659	5827627	IT	-1.01	0.07	5600	3.0	17.3	77.2	5.6	5.4	55	1.7	210	680	224 ± 89	17 ± 3
I50	1870076	5827281	IT	-1.47	0.06	9600	4.5	27.9	68.2	3.9	4.2	34	2	290	920	122 ± 37	13 ± 5
I51	1874915	5828700	IT	2.95	0.52	6700	2.7	3.8	95.7	0.4	1.7	12	1	120	380	116 ± 33	15 ± 2
I52	1871810	5829542	IT	-0.25	0.26	4500	2.7	8.9	89.7	1.4	4.3	20	1	200	450	123 ± 6	17 ± 2
I53	1871371	5827820	SS	0.91	0.33	7500	3.1	9.5	88.9	1.6	2.1	17	1	170	590	258 ± 155	16 ± 4
I54	1873409	5826958	IT	-1.05	0.17	6000	3.4	10.9	87.6	1.5	3.4	24	1	120	350	130 ± 46	17 ± 3
I55	1873681	5825837	IT	-0.95	0.07	16000	3.0	12.6	87.0	0.3	4.3	21	1.1	180	590	136 ± 57	7 ± 1
I56	1874059	5825206	IT	-1.24	0.05	15000	3.3	12.5	87.5	0.1	4.3	35	1.3	130	520	251 ± 150	8 ± 2
I57	1875042	5825703	IT	-1.20	0.06	11000	3.2	6.4	93.5	0.1	2.0	13	1	150	460	83 ± 98	6 ± 1

Site ID	NZTM E	NZTM N	Tidal zone	Depth	Average current speed	Chl-*a*	OM	Mud	Sand	Gravel	Pb	Zn	Cu	TP	TN	N	S
				m	m/s	µg/kg	%	% <63 µm	% ≥63µm <2mm	% ≥2 mm	mg/kg	mg/kg	mg/kg	mg/kg	mg/kg	ind./core	no./core
I58	1876239	5827455	IT	-0.78	0.45	8700	3.5	5.9	88.1	5.9	2.6	22	1	180	410	109 ± 46	16 ± 1
I59	1877894	5826769	IT	-0.99	0.12	4000	1.3	1.8	94.2	4.0	1.6	8	1	91	200	168 ± 41	20 ± 1
I60	1878761	5826878	DS	0.02	0.23	3600	1.8	0.6	99.3	0.1	1.0	11	1	110	190	62 ± 26	8 ± 3
I61	1879047	5826309	IT	-0.61	0.31	8400	2.1	4.0	89.5	6.4	2.3	20	1	180	390	262 ± 31	14 ± 2
I62	1877913	5824841	IT	-1.06	0.05	6600	2.5	12.4	87.2	0.3	2.1	16	1	120	380	46 ± 12	8 ± 1
I63	1878131	5824740	IT	-1.04	0.06	7500	3.1	18.5	81.3	0.4	3.0	45	1.3	180	500	103 ± 88	11 ± 2
I64	1878213	5824451	IT	-1.09	0.06	11000	2.5	5.1	92.7	2.1	1.8	14	1	100	460	61 ± 24	10 ± 2
I65	1880395	5824712	IT	-0.83	0.11	5400	3.2	9.1	90.0	1.0	2.5	22	1.3	160	450	62 ± 22	13 ± 4
I66	1881055	5825407	IT	-0.80	0.09	4100	1.5	4.4	94.7	0.8	1.8	15	1	120	250	54 ± 23	16 ± 4
I67	1882458	5824505	IT	-1.47	0.05	2600	1.9	7.0	92.7	0.4	1.4	10	1	89	220	52 ± 9	13 ± 1
I68	1879334	5822166	IT	-0.77	0.12	9000	2.2	9.5	83.4	7.1	2.5	20	1.7	150	410	83 ± 34	13 ± 2
I69	1878074	5820248	IT	-0.89	0.10	8200	4.0	32.4	63.4	4.2	4.4	44	2.2	210	560	93 ± 15	7 ± 3
I70	1878638	5820282	IT	-1.12	0.13	10000	3.2	21.2	78.2	0.5	3.1	38	1.6	160	280	61 ± 52	9 ± 2
I71	1881779	5821870	IT	0.41	0.37	11000	2.0	2.6	82.8	14.6	1.2	18	1	150	470	209 ± 73	14 ± 1
I72	1883024	5821883	IT	-0.41	0.24	9700	2.4	10.3	88.8	0.9	2.7	20	1.2	190	580	68 ± 21	15 ± 4
I73	1883502	5821744	IT	-0.56	0.17	9800	2.5	14.1	82.0	4.0	2.7	19	1.2	180	640	53 ± 22	11 ± 2

Site ID	NZTM E	NZTM N	Tidal zone	Depth	Average current speed	Chl-*a*	OM	Mud	Sand	Gravel	Pb	Zn	Cu	TP	TN	N	S
				m	m/s	μg/kg	%	% <63 μm	% ≥63μm <2mm	% ≥2 mm	mg/kg	mg/kg	mg/kg	mg/kg	mg/kg	ind./core	no./core
I74	1884604	5822782	IT	-0.94	0.04	9100	1.8	3.5	96.3	0.3	1.2	10	1	93	180	77 ± 16	9 ± 2
I75	1881669	5820495	IT	-0.86	0.18	9100	2.8	12.2	86.9	0.9	2.7	28	1.5	180	280	74 ± 50	11 ± 5
S1	1863119	5849891	SS	-0.81	0.31	13000	1.4	2.9	92.4	4.6	1.7	10	0.4	98	499	84 ± 25	17 ± 2
S2	1863324	5848985	SS	5.02	0.20	13000	2.2	3.3	96.3	0.2	1.9	9	0.4	150	499	65 ± 38	12 ± 4
S3	1861965	5849197	SS	1.64	0.32	23200	1.9	2.6	96.0	1.3	1.6	8	0.4	152	499	63 ± 42	14 ± 3
S4	1860615	5847444	SS	-0.25	0.66	12400	1.9	2.9	87.6	9.3	1.7	8	0.6	87	499	49 ± 16	17 ± 2
S5	1861538	5847070	SS	0.83	0.30	31800	4.2	6.2	93.7	0.1	2.2	12	0.8	167	500	133 ± 42	15 ± 3
S6	1863438	5845776	DS	8.12	0.71	12900	1.4	2.9	84.5	12.6	1.8	8	0.4	109	499	24 ± 11	10 ± 3
S7	1863971	5844105	SS	4.22	0.45	22100	1.5	2.7	93.9	3.3	2.0	8	0.4	119	499	57 ± 11	16 ± 1
S8	1863208	5842925	DS	3.09	0.35	21000	1.8	3.3	94.4	2.4	2.0	10	0.5	118	499	39 ± 19	11 ± 3
S9	1861889	5842056	SS	0.29	0.33	14800	2.0	8.0	91.3	0.8	2.0	10	0.7	114	499	135 ± 55	18 ± 2
S10	1862749	5841987	SS	1.39	0.32	22700	3.2	14.7	83.9	1.5	2.4	12	0.8	141	700	85 ± 32	15 ± 2
S11	1863916	5841646	DS	2.93	0.32	15900	1.8	4.1	89.0	6.9	2.0	9	0.5	110	499	22 ± 3	10 ± 1
S12	1865267	5842276	SS	1.31	0.32	12600	1.8	5.1	94.2	0.8	1.7	9	0.5	79	499	242 ± 54	19 ± 2
S13	1864312	5838107	SS	-0.18	0.37	16300	3.3	12.6	86.9	0.5	2.7	13	1	132	600	525 ± 130	17 ± 2
S14	1865477	5831782	SS	0.47	0.21	22300	3.2	6.2	93.4	0.4	3.2	17	1	153	499	158 ± 13	13 ± 2

Site ID	NZTM E	NZTM N	Tidal zone	Depth	Average current speed	Chl-*a*	OM	Mud	Sand	Gravel	Pb	Zn	Cu	TP	TN	N	S
				m	m/s	µg/kg	%	% <63 µm	% ≥63µm <2mm	% ≥2 mm	mg/kg	mg/kg	mg/kg	mg/kg	mg/kg	ind./core	no./core
S15	1867728	5831371	SS	1.29	0.22	16500	3.3	11.3	85.9	2.8	3.2	19	0.9	154	499	151 ± 58	14 ± 2
S17	1870415	5831000	DS	3.10	0.50	56300	2.1	5.0	86.7	8.3	2.8	19	0.6	115	499	94 ± 72	10 ± 0
S18	1869343	5830086	SS	0.81	0.43	15600	2.7	10.8	87.9	1.2	2.9	20	1	117	499	167 ± 16	18 ± 1
S19	1869104	5828958	SS	0.96	0.27	17600	3.9	18.7	78.4	2.9	3.6	24	1.4	175	600	483 ± 102	18 ± 5
S20	1868665	5827445	SS	0.21	0.31	19200	6.2	22.9	67.4	9.8	6.4	28	3.3	340	1200	484 ± 101	15 ± 1
S21	1870527	5830195	SS	2.67	0.27	19100	3.1	11.9	86.0	2.0	3.2	19	1.2	143	499	367 ± 128	15 ± 3
S22	1869904	5828906	SS	3.32	0.35	14000	2.8	9.2	87.9	2.8	3.2	20	0.9	147	499	323 ± 131	20 ± 1
S23	1869999	5828115	SS	3.13	0.27	8100	3.6	7.9	89.8	2.2	3.3	17	0.8	100	499	429 ± 312	18 ± 3
S24	1870693	5828013	SS	3.49	0.34	12400	4.7	23.4	73.4	3.2	4.6	28	1.9	189	700	188 ± 77	20 ± 5
S25	1872057	5830430	DS	3.56	0.57	39400	1.7	3.4	96.1	0.5	2.0	9	0.5	114	499	94 ± 29	8 ± 4
S26	1872819	5828436	SS	4.44	0.28	8400	3.3	7.9	91.6	0.5	3.7	22	1	187	499	232 ± 148	22 ± 6
S27	1873732	5830110	SS	2.51	0.38	32800	1.8	5.1	90.8	4.2	3.3	20	0.8	111	499	169 ± 44	20 ± 2
S28	1874368	5829059	SS	1.87	0.50	10200	2.7	7.0	80.2	12.8	3.3	16	0.8	180	500	64 ± 24	14 ± 2
S29	1874058	5832821	SS	-0.65	0.33	19100	1.8	6.2	91.5	2.4	2.2	11	0.7	115	500	695 ± 279	17 ± 2
S30	1875880	5830399	DS	-0.16	0.63	34700	1.3	3.0	97.0	0.1	1.7	8	0.4	136	499	118 ± 125	9 ± 4
S31	1876229	5829192	DS	9.00	0.52	2000	1.0	2.6	85.0	12.5	2.1	14	0.5	81	499	33 ± 8	10 ± 3

Site ID	NZTM E	NZTM N	Tidal zone	Depth	Average current speed	Chl-*a*	OM	Mud	Sand	Gravel	Pb	Zn	Cu	TP	TN	N	S
				m	m/s	µg/kg	%	% <63 µm	% ≥63µm <2mm	% ≥2 mm	mg/kg	mg/kg	mg/kg	mg/kg	mg/kg	ind./core	no./core
S32	1876881	5828367	SS	2.39	0.67	22800	2.4	5.3	89.9	5.0	2.4	14	0.9	182	500	131 ± 43	16 ± 1
S33	1878625	5828901	DS	4.91	0.67	2200	1.1	2.4	93.3	4.3	1.4	8	0.3	111	499	86 ± 21	8 ± 1
S34	1875108	5827203	SS	-0.27	0.37	22900	2.1	5.5	91.5	3.0	1.7	10	0.6	104	499	744 ± 654	15 ± 2
S35	1878151	5827359	SS	3.21	0.33	9100	1.3	3.3	90.8	5.9	2.1	12	0.6	134	499	66 ± 6	18 ± 1
S36	1879150	5827089	SS	1.41	0.02	7200	2.2	5.6	81.8	12.7	2.6	15	0.9	85	499	141 ± 28	15 ± 1
S37	1879087	5825602	SS	0.15	0.18	19500	1.9	3.8	85.7	10.4	2.8	19	0.8	91	499	244 ± 215	17 ± 4
S38	1880201	5825764	SS	2.84	0.16	14700	2.9	9.4	89.9	0.7	3.5	20	2.1	182	499	214 ± 127	16 ± 3
S39	1879878	5824854	DS	2.73	0.68	15200	2.4	4.2	78.0	17.8	3.2	22	0.8	108	499	183 ± 96	6 ± 1
S40	1880318	5822864	DS	1.43	0.45	10400	1.7	3.2	87.5	9.4	3.8	25	1	117	499	28 ± 7	7 ± 1
S41	1878919	5820940	SS	-0.53	0.46	41300	2.9	5.0	80.1	15.0	3.0	18	1.4	152	499	218 ± 43	17 ± 4
S42	1880085	5821093	SS	-0.99	0.53	14100	2.0	4.9	88.9	6.2	2.1	28	1.1	117	499	485 ± 176	18 ± 4
S43	1881102	5821482	SS	-0.53	0.29	11200	3.0	14.7	78.5	6.9	6.4	37	3.5	183	500	196 ± 77	13 ± 3
S44	1881346	5821885	SS	0.82	0.36	13000	2.6	8.0	79.6	12.4	5.5	31	2.2	177	499	244 ± 215	17 ± 4
S45	1882682	5822747	SS	-0.29	0.24	14000	2.9	12.3	80.8	7.0	4.2	27	2.7	250	600	303 ± 124	16 ± 2

Abbreviations, Chl-a chlorophyll a, OM organic content, grain size fractions mud, sand and gravel, metals Pb lead, Zn zinc, Cu copper, TP total phosphorous, TN total nitrogen, N average abundance ±SD, S average number of taxa ±SD.

Supplementary Table S2. Summary of measured environmental variables, analysis methods and units.

Variable	Measurement extraction method	Unit
TN	Catalytic combustion, separation, thermal conductivity detector.	mg/kg (dry weight)
TP	Hydrochloric/nitric acid digestion, ICP-MS.	mg/kg (dry weight)
Chl-*a*	Extraction with 95% ethanol, spectroscopy. (NIWA Periphyton Monitoring Manual).	µg/kg
Metals (Cu, Pb, Zn)	Hydrochloric/nitric acid digestion, ICP-MS.	mg/kg (dry weight)
OM	Dry sediment weight loss after combustion at 550°C.	g/100 g
Mud	Grain size < 63 µm	g/100 g
Sand	Grain size ≥ 63 µm, < 2 mm.	g/100 g
Gravel	Grain size ≥ 2mm	g/100 g
Average current speed	Estimated from Estuary Transport Module (Knight, 2019)	m/s
Depth	Elevation model grid corrected to chart datum (CD) (de Ruiter et al. 2019 [56]).	m

TN; total nitrogen, TP; total phosphorous, Chl-a; Chlorophyll-a, Cu; copper, Pb; lead, Zn; zinc, OM; organic content.

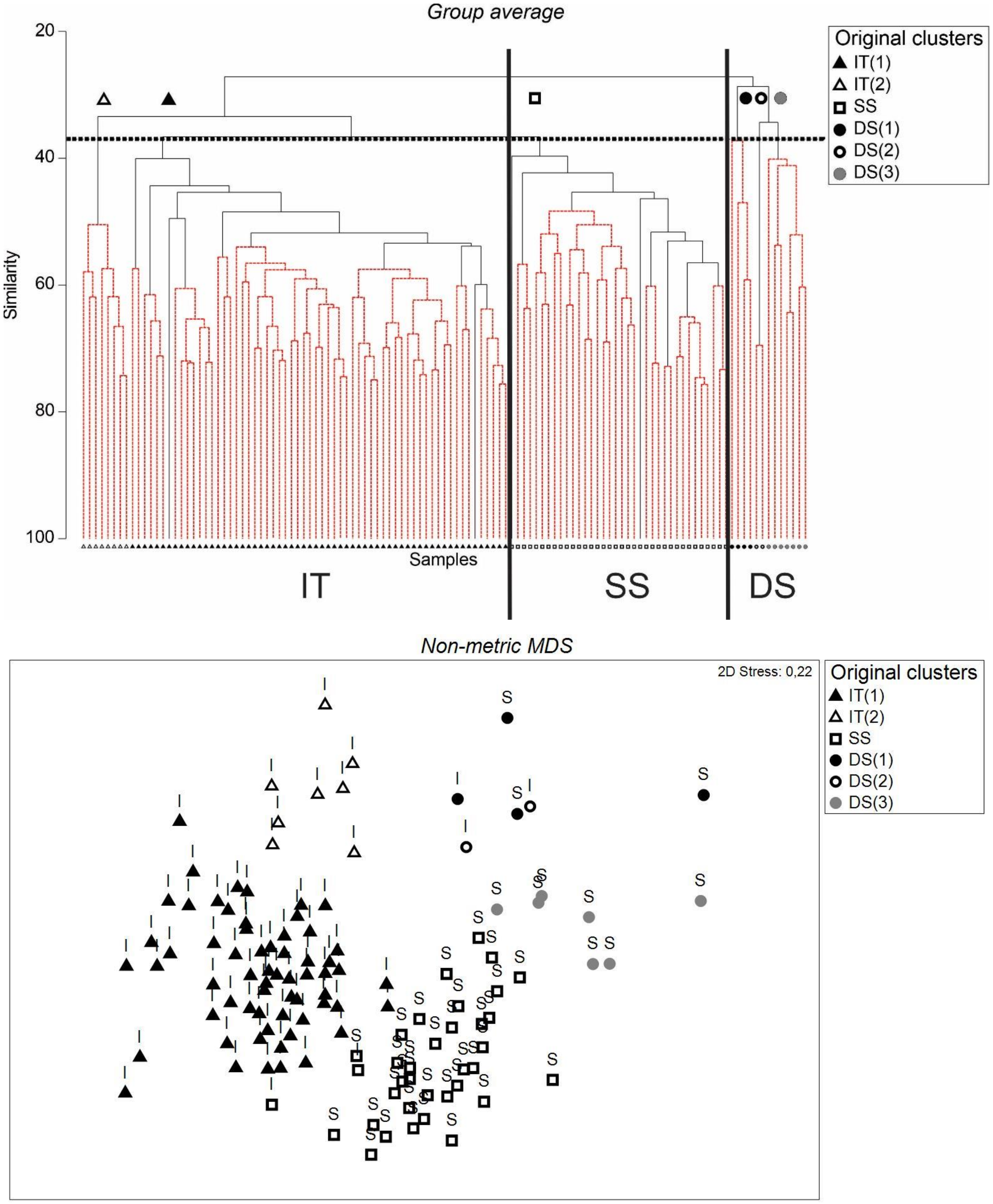

Supplementary Figure S1. Hierarchical cluster analysis with SIMPROF test (red lines indicate non-significant differences) based on the entire taxa abundance data set (square-root transformed, Bray-Curtis similarity) were used to examine the spatial distribution of macrofauna communities, and based on the clusters together with site characteristics, groups were established to represent tidal zones. The black dashed line illustrates the 37% similarity level where six clusters were separated, which are visualised in the non-metric multidimensional scaling (nMDS) plot. The open triangle cluster IT(2) were very shallow and muddy intertidal sites located high up in the estuary and therefore combined with the intertidal cluster (IT(1); black triangles). The shallow subtidal zone is represented by the cluster SS (open squares) and the three other clusters DS(1) DS(2), and DS(3) (black, open, and grey circles) were combined based on site characteristics (e.g. depth, mud content, current speed) and their location in the main channels of the harbour, to represent the deep subtidal zone (DS). Please see Table 2 for site characteristics, and Figure 1 for locations. 'I' and 'S' above symbols indicate within which survey the sites have been sampled, I; intertidal survey 2011-2012, S; subtidal survey 2016.

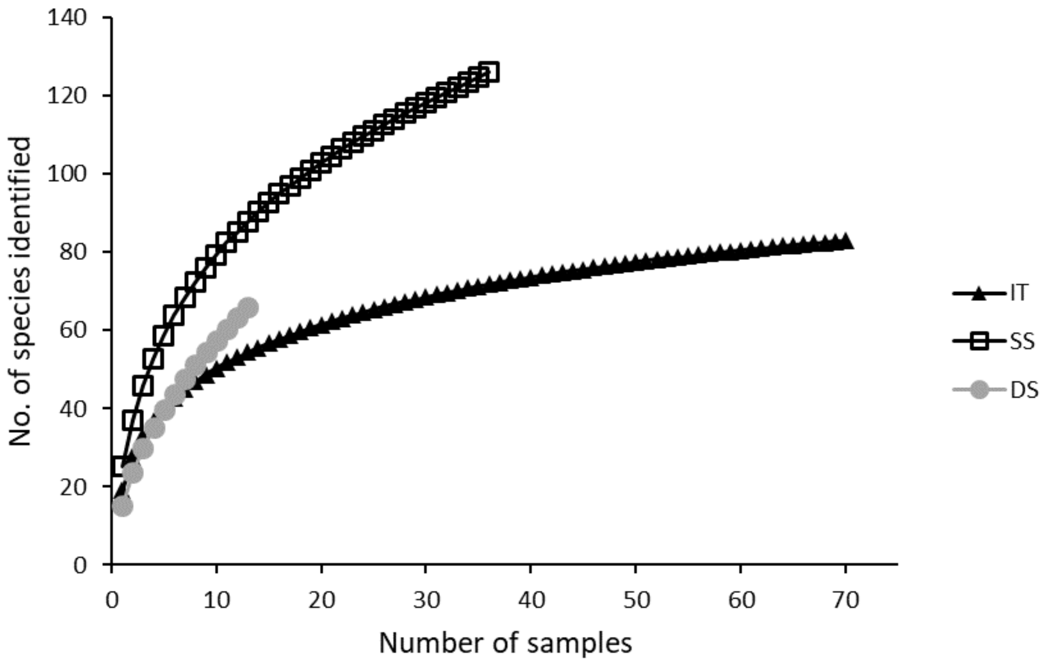

Supplementary Figure S2. Species accumulation curves (SAC) for intertidal (IT; black triangles, n = 70), shallow subtidal (SS; open squares, n = 36) and deep subtidal (DS; grey circles, n =13) sampling sites. To best account for the heterogeneous nature of estuarine communities, SACs were produced using the Ugland, Gray and Ellingsen (UGE) distribution.

Supplementary Table S3. Similarity percentage (SIMPER) analysis results tables summarising key taxa contributing to the dissimilarities observed between a) IT and SS, b) IT and DS, and c) SS and DS macroinvertebrate communities based on square-root transformed macroinvertebrate abundance data. Overall dissimilarity between IT and SS was 66%, IT and DS was 77%, and SS and DS was 72%. Differences between tidal groups are shown to a 40% level. Av. N = average abundance (per core), Av. Diss = average dissimilarity, Diss/SD = ratio of average contribution divided by standard deviation, Contrib. % = contribution percentage, Cum. % = cumulative contribution percentage.

a) Taxa	IT<br>Av. N	SS<br>Av. N	Av. Diss	Diss/SD	Contrib. %	Cum. %
Amphipoda	4.00	4.33	4.53	0.93	6.86	6.86
Spionidae	3.80	5.97	4.53	1.16	6.86	13.73
Aricidea sp.	0.62	3.71	4.18	1.24	6.34	20.06
Oligochaeta	2.11	4.34	3.67	0.90	5.57	25.63
Heteromastus filiformis	2.77	3.84	3.23	1.26	4.90	30.53
Syllidae	0.95	3.01	3.11	1.25	4.71	35.24
Paraonidae	0.28	2.57	2.90	1.43	4.39	39.64
Linucula hartvigiana	1.98	0.68	2.16	1.12	3.28	42.92

b) Taxa	IT<br>Av. N	DS<br>Av. N	Av. Diss	Diss/SD	Contrib. %	Cum. %
Spionidae	3.80	0.69	5.81	1.73	7.51	7.51
Amphipoda	4.00	2.87	5.64	0.96	7.29	14.80
Paphies australis	0.08	2.43	4.50	0.62	5.82	20.61
Heteromastus filiformis	2.77	0.76	4.21	1.13	5.44	26.06
Linucula hartvigiana	1.98	0.00	3.35	1.12	4.33	30.39
Hesionidae	0.01	1.73	3.25	0.91	4.21	34.60
Austrovenus stutchburyi	1.92	0.04	3.08	1.05	3.99	38.58
Oligochaeta	2.11	0.94	2.92	1.00	3.78	42.36

c) Taxa	SS<br>Av. N	DS<br>Av. N	Av.Diss	Diss/SD	Contrib. %	Cum. %
Spionidae	5.97	0.69	7.17	1.38	10.00	10.00
Oligochaeta	4.34	0.94	4.77	1.04	6.65	16.66
Aricidea sp.	3.71	0.39	4.70	1.24	6.56	23.22
Heteromastus filiformis	3.84	0.76	4.50	1.48	6.28	29.50
Amphipoda	4.33	2.87	4.37	0.90	6.09	35.59
Paphies australis	0.63	2.43	3.75	0.71	5.23	40.82

Supplementary Table S4. Summary of one-way PERMANOVA comparing functional group abundance data (square-root transformed data, Bray-Curtis similarity matrix) between tidal zones (Intertidal, IT; Shallow subtidal, SS; Deep subtidal, DS).

Source	df	SS	MS	Pseudo-F	P(perm)	Post-hoc pairwise tests
Tidal zone	2	29031	14515	15.093	<0.001	I ≠ SS ≠ SD
Residuals	116	1.1156×10^5	961.71			
Total	118	1.4059×10^5				

Supplementary Table S5. Similarity percentage (SIMPER) analysis results table summarising key functional groups (FG) contributing to the dissimilarities observed between a) IT and SS, b) IT and DS, and c) SS and DS macroinvertebrate communities based on square-root transformed functional group abundance data. Overall dissimilarity between IT and SS was 51%, IT and DS was 58%, and SS and DS was 56%. Differences between tidal groups are shown to a 70% level. Av. N = average abundance (per core), Av. Diss = average dissimilarity, Diss/SD = ratio of average contribution divided by standard deviation, Contrib. % = contribution percentage, Cum. % = cumulative contribution percentage. For corresponding functional group attributes see Table 1.

a) Functional group	IT Av. N	SS Av. N	Av. Diss	Diss/SD	Contrib. %	Cum. %
FG 13	3.15	6.67	6.24	1.42	12.13	12.13
FG 22	4.01	4.33	5.47	0.95	10.63	22.76
FG 12	3.95	6.05	5.41	1.17	10.51	33.27
FG 17	1.21	4.36	5.10	1.61	9.91	43.18
FG 19	2.12	4.45	4.51	0.95	8.78	51.96
FG 2	1.94	1.09	2.73	1.13	5.31	57.26
FG 6	2.09	0.74	2.72	1.15	5.30	62.56
FG 23	1.49	2.41	2.42	0.99	4.70	67.26
FG 5	1.62	0.51	1.97	1.31	3.82	71.08
**b) Functional group**	**IT Av. N**	**DS Av. N**	**Av. Diss**	**Diss/SD**	**Contrib. %**	**Cum. %**
FG 12	3.95	0.87	6.77	1.67	11.70	11.70
FG 22	4.01	2.91	6.46	0.99	11.16	22.86
FG 2	1.94	2.52	6.26	0.93	10.82	33.68
FG 13	3.15	3.03	4.77	1.32	8.25	41.92
FG 19	2.12	2.19	4.19	1.20	7.24	49.17
FG 6	2.09	0.04	4.16	1.18	7.20	56.37
FG 17	1.21	1.84	3.24	1.23	5.61	61.98
FG 8	1.51	0.09	3.13	1.91	5.41	67.38
FG 5	1.62	0.20	3.11	1.44	5.37	72.76
**c) Functional group**	**SS Av. N**	**DS Av. N**	**Av. Diss**	**Diss/SD**	**Contrib. %**	**Cum. %**
FG 12	6.05	0.87	8.67	1.39	15.54	15.54
FG 13	6.67	3.03	6.85	1.49	12.28	27.83
FG 22	4.33	2.91	5.36	0.92	9.62	37.44
FG 19	4.45	2.19	5.29	1.02	9.49	46.94
FG 2	1.09	2.52	4.81	0.80	8.64	55.57
FG 17	4.36	1.84	4.79	1.54	8.60	64.17
FG 23	2.41	0.85	2.94	1.01	5.28	69.45
FG 20	1.09	0.13	1.85	0.98	3.33	72.78
